# GWAS with Heterogeneous Data: Estimating the Fraction of Phenotypic Variation Mediated by Gene Expression Data

**DOI:** 10.1534/g3.118.200571

**Published:** 2018-08-01

**Authors:** Eriko Sasaki, Florian Frommlet, Magnus Nordborg

**Affiliations:** * Gregor Mendel Institute, Austrian Academy of Sciences, Vienna B(VBC), Vienna, Austria; † Center for Medical Statistics, Informatics, and Intelligent Systems, Medical University Vienna, Vienna, Austria

**Keywords:** mediation analysis, flowering time, natural variation, FLC, genetic architecture, correlation network

## Abstract

Intermediate phenotypes such as gene expression values can be used to elucidate the mechanisms by which genetic variation causes phenotypic variation, but jointly analyzing such heterogeneous data are far from trivial. Here we extend a so-called mediation model to handle the confounding effects of genetic background, and use it to analyze flowering time variation in *Arabidopsis thaliana*, focusing in particular on the central role played by the key regulator *FLOWERING TIME LOCUS C* (*FLC*). *FLC* polymorphism and *FLC* expression are both strongly correlated with flowering time variation, but the effect of the former is only partly mediated through the latter. Furthermore, the latter also reflects genetic background effects. We demonstrate that it is possible to partition these effects, shedding light on the complex regulatory network that underlies flowering time variation.

A crucial question in genetics is understanding how genetic variation translates into phenotypic variation. DNA sequence polymorphisms influence final phenotypes through intermediate phenotypes such as protein structures, epigenetic states, and gene expression levels—many of which can be assayed using modern technologies. Understanding how these intermediate, molecular phenotypes mediate the effects of genetic variation is of fundamental interest, and has enormous applied implications.

Interest has in particular focused on gene expression levels since they dynamically respond to environmental stimuli, developmental transitions, and other physiological states. Mapping studies have shown that eQTL (expression Quantitative Trait Loci) frequently coincide with causal variants identified using GWAS (Genome-Wide Association Studies, see; Nicolae *et al.* 2010; GTEx Consortium 2017), supporting the notion that a substantial proportion of genetic variants influence the phenotype by regulating expression levels of the corresponding genes (Nicolae *et al.* 2010; Cubillos *et al.* 2012; Barfield *et al.* 2017; Chun *et al.* 2017; Mancuso *et al.* 2017). However, even if this were true, the correlation between *measured* expression variation and phenotypic variation would not necessarily be perfect due to time-, tissue-, and environment-specific regulation. To quantify this, the genetic effect can be decomposed using a “mediation model” (see Materials and Methods for more about medation models) into an “indirect effect” that can be explained by gene expression levels and a “direct effect” that cannot be (Figure 1; Baron 1986; Valeri and Vanderweele 2013; Huang *et al.* 2015). A recent study reported that only about 20% of human disease heritability is thus mediated by gene expression (O’Connor *et al.* 2017).

**Figure 1:**
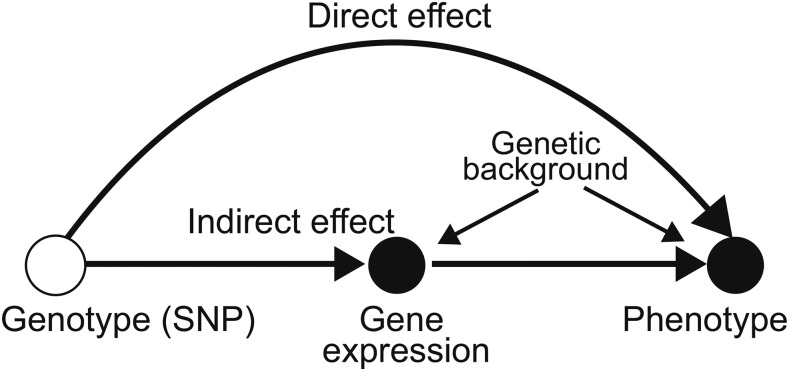
A genotype-phenotype model that includes gene expression. The phenotype is affected by a genetic polymorphism that is partly mediated by the expression of a nearby gene, resulting in a direct and indirect genetic effect. Both gene expression and phenotype are also affected by confounding genetic background.

Here we use GWAS and mediation analysis to study the transcriptional network regulating flowering time in *Arabidopsis thaliana*. A novel feature of our analysis is that we explictly model the confounding effects of the genetic background using a linear mixed-model approach that has become standard in GWAS (Vilhjálmsson and Nordborg 2013). That confounding can bias mediation analyses is well known (Richiardi *et al.* 2013; Yang *et al.* 2017), as is the fact that genetic background is a major confounder of GWAS in *A. thaliana* — especially of locally adaptive traits (Aranzana *et al.* 2005; Atwell *et al.* 2010). To our knowledge this is the first time that mediation analysis including random effects for the genetic background is performed, where we justify our approach using the statistical theory of counterfactuals.

Flowering time in *A. thaliana* is well-suited for the development of mediation models for at least three reasons. First, systematically collected multi-layer data are available. Since *A. thaliana* is highly selfing and naturally exists as inbred lines, multiple phenotypes, including intermediate ones such as gene expression, have been collected for the same genotype. Second, sampling and growth conditions are controllable and uniform, unlike in human studies, making modeling easier. Finally, flowering time is one of the best understood traits in plants. More than one hundred genes in several major pathways have been described: the photoperiod, ambient temperature, autonomous, integrator, gibberellin and vernalization pathways combine to regulate flowering (Simpson and Dean 2002; Kim *et al.* 2009; Wellmer and Riechmann 2010; Srikanth and Schmid 2011; Andrés and Coupland 2012).

Our primary goal in this study is to use flowering time as an example to explore how best to combine heterogeneous, multilayer data in order to improve our understanding of the genotype-phenotype map. Our results illustrate well the complexities inherent in even a very simple network structure.

## Materials and Methods

### Data sets

We used published *A. thaliana* data sets containing genotypes (Long *et al.* 2013), RNA-seq transcriptome data (Dubin *et al.* 2015), as well as flowering time phenotypes (Sasaki *et al.* 2015, Table S1). All plants were grown under constant 10° (132 lines) and 16° (154 lines) in 16 h day length condition. For RNA seq analysis, RNA was extracted from whole rosettes collected at 11-12 h after dawn at nine-leaf stage (Dubin *et al.* 2015). In addition, we used a dataset for flowering time and *FLC* expression including global populations (Shindo *et al.* 2005; Atwell *et al.* 2010, 101 lines). Plants were grown under natural light conditions in the greenhouse (22-23°) from October 2002 to March 2003. *FLC* expression was measured by q-RT-PCR using RNA extracted from young leaves after 4 weeks of growth (nearly nine-leaf stage). With respect to genotypes the genome-wide SNP information in the 1001 genome project was used (The 1001 Genomes Consortium 2016). The dataset of 10° were used for model building, it of 16° and greenhouse were used for prediction of flowering time by the model.

### Correlation analysis

**Figure 2:**
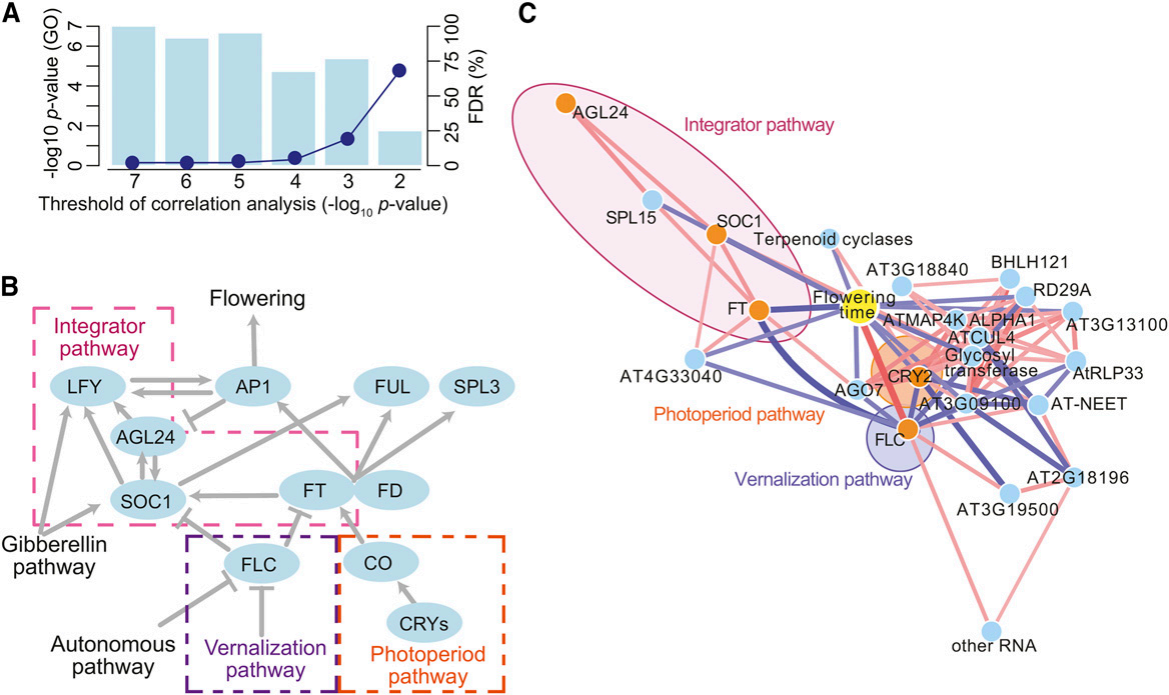
Correlation between flowering time and gene expression levels in the Swedish population. (A) The significance of the GO enrichment for flowering time genes (and implied FDR; see Methods) as function of the significance threshold for the flowering-expression correlation. (B) Outline of the flowering pathways in *A. thaliana* (reviewed in, *e.g.*, Kim *et al.* 2009; Wellmer and Riechmann 2010; Srikanth and Schmid 2011). *FLC* represses the floral integrator genes *FD*, *FT*, and *SOC1*. *FT* is induced by the photoperiod pathway through *CONSTANS* (*CO*), which is induced by *CRYPTOCROMEs* (*CRYs*); the *FT* protein is a mobile flowering signal that works with *FD* to induce *SOC1* and floral meristem genes including *APETALA1* (*AP1*), *FRUITFUL* (*FUL*), and *SEPALATA* (*SPL3*). *AGL24* and *SOC1* regulate each other in positive feedback loops and induce transcription of *LFY*. The gibberellin pathway promotes flowering by inducing *SOC1* and the floral meristem-identity gene *LEAFY* (*LFY*). (C) A correlation network based on gene expression levels. Nodes show flowering time (yellow) and the genes in Table 1 (blue, or orange for the *a priori* gene set). Edges show significant correlations between nodes (with Bonferroni correction to control FWER at α=0.01) in pink or blue (for positive and negative correlations, respectively).

Both Spearman’s (*ρ*) and Pearson’s (*r*) correlation coefficient between flowering time and expression levels were calculated for 20,285 genes for which more than 10% lines showed detectable expression levels. The Benjamini Hochberg prodecure (Benjamini 1995) was applied to the *p*-values corresponding to *ρ* to obtain genes with the most highly correlated expression levels while controlling FDR at 5%. For the resulting genes a correlation network (Figure 2) was visualized with Cytoscape (Shannon *et al.* 2003) using a Bonferroni corrected threshold of 1.35e-05 for *p*-values ($741=\left(\begin{array}{c} 39\\ 2 \end{array}\right)$ tests for pairs of 38 genes + flowering time at a family wise error rate of 0.01).

### GO analysis

Enrichment of known flowering time genes was estimated using BiNGO as a plugin of Cytoscape (Maere *et al.* 2005) with Benjamini-Hochberg FDR correction (Benjamini 1995). The GO term ”regulation of flower development” (TAIR; Berardini *et al.* 2015) was used for the analysis of flowering time genes. FDR was calculated based on the GO list as described in Sasaki *et al.* (2015).

### Genome-Wide Association Studies (GWAS)

GWAS analysis for flowering time and *FLC* expression was performed using LIMIX (Lippert *et al.* 2014), and the following liner mixed-model (LMM):

Y=Xβ+u+e

$$\mathrm{var}\left(Y\right)=\sigma_{g}^{2}K+\sigma_{e}^{2}I$$

where Y is the n×1 vector of a phenotype (either the standardized flowering time or the standardized *FLC* expression), X is the n×1 vector of the standardized genotype to be tested (SNP), and *β* is the parameter of the corresponding fixed effect. $u∼N\left(0,\sigma_{g}^{2}K\right)$ is the random effect including the kinship matrix K representing genetic relatedness (IBS) (Yu *et al.* 2006; Kang *et al.* 2008) and $e∼N\left(0,\sigma_{e}^{2}I\right)$ refers to the residual. Bonferroni-correction was used for multiple-testing correction (using a family wise error rate of 5% with 3,401,897 SNPs after excluding all SNPs with MAF ≤0.1).

### Variance component analysis

*Cis*-genetic effects of loci on an expression level **Y** was estimated using local_vs_global_mm() function in mixmogam (https://github.com/bvilhjal/mixmogam) with the model

$$Y=U_{\text{local}}+U_{\text{global}}+\varepsilon U_{\text{local}}∼N\left(0,\sigma_{\text{local}}^{2}K_{\text{local}}\right)U_{\text{global}}∼N\left(0,\sigma_{\text{global}}^{2}K_{\text{global}}\right)\varepsilon_{Y}∼N\left(0,\sigma_{y}^{2}I_{\text{n}}\right)$$

Here $U_{\text{local}}$ and $U_{\text{global}}$ are random effects corresponding to local and global relatedness, respectively, and *ε* is noise. The local region is defined as ± 15 Kb coding region of each gene, and the global region is defined as the entire genome. With mixmogam the local and global IBS matrices were calculated as genetic relatedness using all SNPs in local and global regions, respectively. Significance of the variance components was estimated by permutation tests (1000 times) with maintaining the chromosomal order of all observations but shuffling the relative positions of the two variables.

### Mediation analysis

The idea of using pathway analysis to dissect biological effects into direct and indirect causal relationships was developed already about 100 years ago by Wright (1921). However, his methods were by and large ignored in the biological sciences (see Shipley 2016, for a discussion of why this was the case), and it was rather in the social sciences that similar ideas were developed almost half a century later. For example, Baron and Kenny (1986) discussed questions of mediation analysis in the context of pathway models. The modern approach to causal inference however relies upon the counterfactual framework (see, for example: Pearl 2009; Imbens and Rubin 2015).

To develop these ideas denote by *X* some input variable, by *M* the mediator and by *Y* the outcome. In the counterfactual framework one conceptualizes for each individual different potential outcomes depending on the state of other variables. For example one would denote by $Y_{x}\left(u\right)$ the state of *Y* for individual *u* when *X* would be equal to *x*. Although in practice never observable, one contemplates the potential outcomes depending on different values of *x* as mathematically existing entities — the counterfactual variables. The average causal effect (or total effect) of a change from *x* to $x^{*}$ is then defined by $E\left(Y_{x}-Y_{x^{*}}\right).$ Under certain assumptions it is then possible to estimate this causal effect from observational data (Pearl 2009; VanderWeele 2015).

If one is interested in the effect that changing *x* has on the outcome *Y* directly (*i.e.*, effects that are not mediated by other variables), then the first idea is to look at counterfactual outcomes when keeping the levels *m* of the mediator *M* fixed. This leads to the so-called Controlled Direct Effect $\text{CDE}=E\left(Y_{xm}-Y_{x^{*}m}\right).$ The CDE is not very appealing in our case for two reasons. First, the gene expression levels (our mediator) can certainly not be controlled at all, and, second, controlling does not provide the definition of an indirect effect. In contrast, the concepts of natural direct effect (NDE) and natural indirect effect (NIE) introduced by Pearl (2001) are directly applicable. The natural direct effect defined as $\text{NDE}=E\left(Y_{xm_{x^{*}}}-Y_{x^{*}}\right)$ compares (at an individual level) the change in the outcome *Y* between input $x^{*}$ and input *x* but assuming the mediator level would take the counterfactual value $m_{x^{*}}.$ So NDE measures the change in outcome when the mediator level is kept fixed while changing input *x*. In contrast the natural indirect effect is defined as $\text{NIE}=E\left(Y_{x^{*}m_{x}}-Y_{x^{*}}\right),$ so here the input is kept fixed at $x^{*}$ and one measures the change in outcome that would occur by changing the mediator according to the counterfactual $m_{x}.$ Identifiability assumptions for NDE and IDE for observational data are given in Pearl (2001) and VanderWeele and Vansteelandt (2009).

Now the concepts of CDE, NDE and IDE make it possible to obtain clear definitions of direct and indirect effects for rather general classes of regression models. We want to illustrate this first in the context of the simplest possible linear regression model for mediation analysis. To this end consider data from a sample of size *n* with input $X\in {\mathbb{R}}^{n},$ a mediator $M\in {\mathbb{R}}^{n}$ and a trait variable $Y\in {\mathbb{R}}^{n}$ and consider the model

$$M=X\beta_{1}+\varepsilon_{M},\varepsilon_{M}∼N\left(0,\sigma_{M}^{2}I_{n}\right)Y=X\beta_{2}+M\theta_{1}+\varepsilon_{Y},\varepsilon_{Y}∼N\left(0,\sigma_{Y}^{2}I_{n}\right),$$

where $I_{n}$ is the n×n identity matrix, $\beta_{1}$ and $\beta_{2}$ are parameters for the fixed effects, and $\theta_{1}$ is the parameter for the effect of the mediator *M*. In our case, *X* corresponds to SNP${}_{FLC}$, *M* to the gene expression levels of the corresponding gene and *Y* denotes the flowering time. The classical approach of pathway analysis simply consists of plugging in the model for *M* in the second equation for *Y*,

$$Y=X\beta_{2}+\left(X\beta_{1}+\varepsilon_{M}\right)\theta_{1}+\varepsilon_{Y},$$ (1)

with corresponding expectation

$$E\left(Y\right)=X\left(\beta_{2}+\beta_{1}\theta_{1}\right).$$ (2)

Then $\beta_{2}$ would be referred to as the direct effect and $\beta_{1}\theta_{1}$ as the indirect effect which is mediated through *M*. It turns out that for the simple model (1) both CDE and NDE coincide with the direct effect from pathway analysis $\text{CDE}=\text{NDE}=\beta_{2}$ and also $\text{NIE}=\beta_{1}\theta_{1}.$ This follows for example from the analysis given in VanderWeele and Vansteelandt (2009), where a slightly more general model including interactions between the input *X* and the mediator *M* is considered. For the linear model (1) standard software for regression can then be used to obtain estimates of NDE and NIE and VanderWeele and Vansteelandt (2009) also show how to compute the corresponding standard deviations. A simple SAS macro to perform these computations is described in Valeri and Vanderweele (2013).

One shortcoming of this extremely simple mediation approach is that it does not take into account at all the polygenic effect from other SNPs. The customary mixed model approach to GWAS analysis uses a random effect to model that polygenic effect and we would like to incorporate such random effects into the mediation analysis. Thus consider the following generalization of (1)

$$M=X\beta_{1}+Z\gamma_{1}+\varepsilon_{M},\varepsilon_{M}∼N\left(0,\sigma_{M}^{2}I_{n}\right)$$ (3)

$$Y=X\beta_{2}+M\theta_{1}+Z\gamma_{2}+\varepsilon_{Y},\varepsilon_{Y}∼N\left(0,\sigma_{Y}^{2}I_{n}\right),$$ (4)

where $\gamma_{1}$ and $\gamma_{2}$ act as random effects for the polygenic effects by all other SNPs, say $Z=\left(X_{1},\ldots ,X_{p}\right)$ (where the SNP genotypes have been standardized) and $\gamma_{i}∼N_{p}\left(0,\sigma_{i}^{2}I_{p}\right).$ Now the error terms $\varepsilon_{M}$ and $\varepsilon_{Y}$ might be seen specifically to model environmental effects and measurement errors of *M* and *Y*, respectively. According to the pathway approach plugging in the model for *M* in the second equation for *Y* now yields

$$Y=X\beta_{2}+\left(X\beta_{1}+Z\gamma_{1}+\varepsilon_{M}\right)\theta_{1}+Z\gamma_{2}+\varepsilon_{Y},$$ (5)

and taking expectations again results in (2). Therefore, according to pathway analysis the definitions of direct and indirect effects remain exactly the same as in case of the standard mediation model (1) without random effects. In terms of counterfactuals it is straight forward to see that

$$\text{NDE}=E\left(Y_{xm_{x^{*}}}-Y_{x^{*}}\right)=E\left(Y_{xm_{x^{*}}}-Y_{x^{*}m_{x^{*}}}\right)=\beta_{2}x+\theta_{1}m^{*}-\beta_{2}x^{*}-\theta_{1}m^{*}=\beta_{2}\left(x-x^{*}\right)$$

and denoting by $f_{M|X}\left(m|x\right)$ the conditional density function of *M* given X=x we obtain

$$\text{NIE}=E(Y_{x^{*}m_{x}}-Y_{x^{*}})=E(Y_{x^{*}m_{x}}-Y_{x^{*}m_{x^{*}}})=\int_{m}E\left(Y|x^{*},m_{x}\right)f_{M|X}\left(m|x\right)dm-\int_{m}E\left(Y|x^{*},m_{x^{*}}\right)f_{M|X}\left(m{|x}^{*}\right)dm=\int_{m}\left(x^{\text{*}}\beta_{2}+m\theta_{1}\right)f_{M|X}\left(m|x\right)dm-\int_{m}\left(x^{*}\beta_{2}+m\theta_{1}\right)f_{M|X}\left(m|x^{*}\right)dm=\theta_{1}E\left(M|X=x\right)-\theta_{1}E\left(M|X=x^{*}\right)=\theta_{1}\beta_{1}\left(x-x^{\text{*}}\right)$$ (6)

where we used (4) for the fourth equality and (3) for the last equality. In summary, it follows that also in case of the mixed model, the direct and indirect effects based on counterfactuals coincide with the effects already obtained for the simple linear model. The only remaining question is how to efficiently estimate the parameters $\beta_{1},\beta_{2}$ and $\theta_{1}.$ This problem has been comprehensively studied and a number of software packages are available (*e.g.*, Kang *et al.* 2008).

Using the notation $K=ZZ^{\prime }$ for the kinship matrix we obtain

$$\begin{array}{l} \mathrm{Var}\left(M|X\right)=\sigma_{1}^{2}K+\sigma_{M}^{2}I_{n}=\sigma_{M}^{2}\left(\lambda_{1}K+I_{n}\right)\\ \mathrm{Var}\left(Y|M\right)=\sigma_{2}^{2}K+\sigma_{Y}^{2}I_{n}=\sigma_{Y}^{2}\left(\lambda_{2}K+I_{n}\right) \end{array}$$

with $\lambda_{1}=\sigma_{1}^{2}/\sigma_{M}^{2}$ and $\lambda_{2}=\sigma_{2}^{2}/\sigma_{Y}^{2}.$ The same software packages can be used to estimate these ratios (Kang *et al.* 2008). Scripts used for the mediation analysis are available in supplemental scripts.

To test whether there is an indirect effect, that is the null hypothesis *β*_1_=0, we used permutation tests. Gene expression values were permuted 1500 times while keeping flowering time, genotype and the relatedness matrix fixed.

### Estimation of explained variance

The amount of flowering time variation explained by SNP${}_{FLC}$ and *FLC* expression was estimated using the $r^{2}$ defined for the LMM by Nakagawa and Schielzeth (2013). We estimated $r^{2}$ for three models:

- $r_{\text{total}}^{2},$ for the full model including SNP and expression effects $Y=X\beta_{2}+M\theta_{1}+Z\gamma_{2}+\varepsilon_{Y}$ given in equation (4);
- $r_{\text{SNP}}^{2},$ for a SNP model $Y=X\beta +Z\gamma +\varepsilon_{Y},$ and;
- $r_{\text{expression}}^{2},$ for a SNP-independent expression model $Y=M\theta +Z\gamma +\varepsilon_{Y}$ (estimated as $r_{\text{expression}}^{2}=r_{\text{total}}^{2}-r_{\text{SNP}}^{2}$).

### Prediction of flowering time

Flowering time was predicted using the full mediation model given by equation (4), using estimates of $\beta_{2}$ and $\theta_{1}$ from the 10° data $\left(\beta_{2}=0.25,\;\;\theta_{1}=0.51\right).$ Based on the assumption that effects of population structure on Y and M are proportional, we then estimate $Z\gamma_{2}$ for each new data set by fitting a null model $M=Z\gamma_{2}+\varepsilon_{M}$ by REML as implemented in EMMA (Kang *et al.* 2008). The variation explained by the resulting model was estimated using $r^{2}$ as just described (Nakagawa and Schielzeth 2013). To test whether the variance component was positive permutation tests were applied (Figure S2).

### Data availability

Table S2 contains all flowering time and FLC expression data. Other gene expression data (Dubin *et al.* 2015) are available at GEO with accession GSE54680. SNP data sets are available at https://github.com/Gregor-Mendel-Institute/swedishgenomes (Long *et al*. 2013) and http://1001genomes.org (The 1001 Genomes Consortium 2016). All scripts used for the mediation analysis are available in supplemental scripts. Supplemental material available at Figshare: https://doi.org/10.25387/g3.6837674.

## Results

### Correlation between gene expression and flowering time

We began by asking whether gene expression, as measured in whole plants (above-ground tissue only) at a few weeks of age (the nine-leaf stage) was correlated with the eventual flowering of the same genotype (at 10° under long-day conditions; see Methods) across 132 inbred lines (Table S1). According to the Benjamini Hochberg procedure at an FDR level of 5%, 38 out of 20,285 genes (0.2%) showed significant correlation with flowering time (Table 1). Of these, 9 were annotated as being related to flowering, and 5 were also part of a more conservative list of *a priori* candidates (Srikanth and Schmid 2011). This represents a highly significant enrichment, which persists at higher FDR cut-offs (Figure 2A; see Methods).

**Table 1:** List of genes whose expression is significantly correlated with flowering time. Spearman correlation coefficient *ρ* with its corresponding *p*-value, as well as the squared Pearson correlation coefficient $r^{2}$ which quantifies the explained variation of a simple linear model

Gene ID	*ρ*	*p*-value	$r^{2}$	Description*^a^*
AT5G10140	0.63	3.05E-16	0.53	FLC*
AT1G65480	−0.54	2.64E-11	0.37	FT*
AT2G45660	−0.47	1.35E-08	0.22	SOC1*
AT2G41640	−0.42	7.03E-07	0.20	Glycosyl-
				transferase
AT3G57920	−0.39	3.28E-06	0.17	***SPL15***
AT1G04400	−0.38	5.24E-06	0.15	CRY2*
AT5G52310	−0.38	5.39E-06	0.14	*RD29A*
AT1G69440	−0.38	5.53E-06	0.18	*AGO7*
AT3G13100	−0.38	7.71E-06	0.10	ATP-BINDING
				CASSETTE C7
AT1G23870	−0.38	8.98E-06	0.16	*TPS9*
AT5G44630	−0.37	9.65E-06	0.13	Terpenoid cyclases
AT3G09100	−0.37	9.74E-06	0.11	protein coding
AT5G51720	0.37	9.90E-06	0.07	***AT-NEET***
AT4G33040	−0.37	1.02E-05	0.12	protein coding
AT3G04485	0.37	1.51E-05	0.13	other RNA
AT1G77810	−0.37	1.62E-05	0.10	Galactosyl-
				transferase
AT2G13560	−0.36	1.70E-05	0.11	*NAD-ME1*
AT3G08990	0.36	1.73E-05	0.08	protein coding
AT1G17020	−0.36	1.78E-05	0.07	*SRG1*
AT1G06160	0.36	2.26E-05	0.07	*ORA59*
AT3G19860	−0.36	2.35E-05	0.11	BHLH121
AT5G48400	−0.36	2.60E-05	0.10	*ATGLR1.2*
AT3G19500	0.36	2.76E-05	0.14	protein coding
AT3G05660	−0.36	2.80E-05	0.11	*AtRLP33*
AT4G24540	−0.35	3.33E-05	0.11	AGL24*
AT5G25120	−0.35	3.42E-05	0.15	*CYP71B11*
AT3G18840	−0.35	4.03E-05	0.08	TPR-like super-
				family protein
AT2G18196	0.35	4.67E-05	0.11	protein coding
AT5G46210	−0.35	4.78E-05	0.10	***ATCUL4***
AT1G53165	−0.35	5.01E-05	0.09	ATMAP4K
				ALPHA1
AT3G20250	−0.34	5.12E-05	0.09	*APUM5*
AT5G44590	0.34	5.68E-05	0.12	protein coding
AT3G55610	−0.34	6.47E-05	0.12	*P5CS2*
AT4G18130	−0.34	6.63E-05	0.13	***PHYE***
AT1G78050	−0.34	6.82E-05	0.12	*PGM*
AT5G10490	−0.34	6.94E-05	0.12	*MSL2*
AT5G58900	0.34	7.22E-05	0.10	protein coding
AT2G46500	−0.34	7.92E-05	0.11	*ATPI4K*

a Genes in bold have flowering-related mutant phenotypes; *denotes genes that are also part of a more conservative list of *a priori* candidates (Srikanth and Schmid 2011).

The top three genes (Table 1) were all *a priori* flowering time genes: *FLOWERING LOCUS C* (*FLC*; Michaels and Amasino 1999; Sheldon *et al.* 1999) in the vernalization pathway, and *FLOWERING LOCUS T* (*FT*; Kardailsky *et al.* 1999; Kobayashi *et al.* 1999) and *SUPPRESSOR OF OVEREXPRESSION OF CONSTANS 1* (*SOC1*; Samach *et al.* 2000) in the ”integrator” pathway (Table 1; Figure 2B). In agreement with previous work, *FLC* expression was clearly most strongly correlated: the explained variance, $r^{2}$ = 0.53, is strikingly similar to what was seen by Lempe *et al.* (2005) using a sample under different environmental conditions. The expression of the integrator loci *FT* and *SOC1* is less strongly correlated with flowering, which is interesting given that these loci are thought to act downstream of *FLC*, and are in this sense closer to the phenotype (Figure 2B; Schmid *et al.* 2003; Wellmer and Riechmann 2010).

The correlation network connecting the genes in Table 1 with flowering was consistent with the known flowering-time pathways (Figure 2C). The integrator pathway connected *FT* and *SOC1* with another strong *a priori* candidate, *AGL24*, a known inducer of *SOC1* (Yu *et al.* 2002, 2004; Michaels *et al.* 2003). The photoperiod pathway was not connected with the integrator pathway but included *CRY2* (Toth *et al.* 2001) as a hub gene in a network containing 19 other genes. The vernalization pathway, via *FLC*, cleary plays a central role, connecting the integrator pathway and the photoperiod pathways via *FT* and *CRY2*.

### The genetic basis of expression and flowering variation

**Figure 3:**
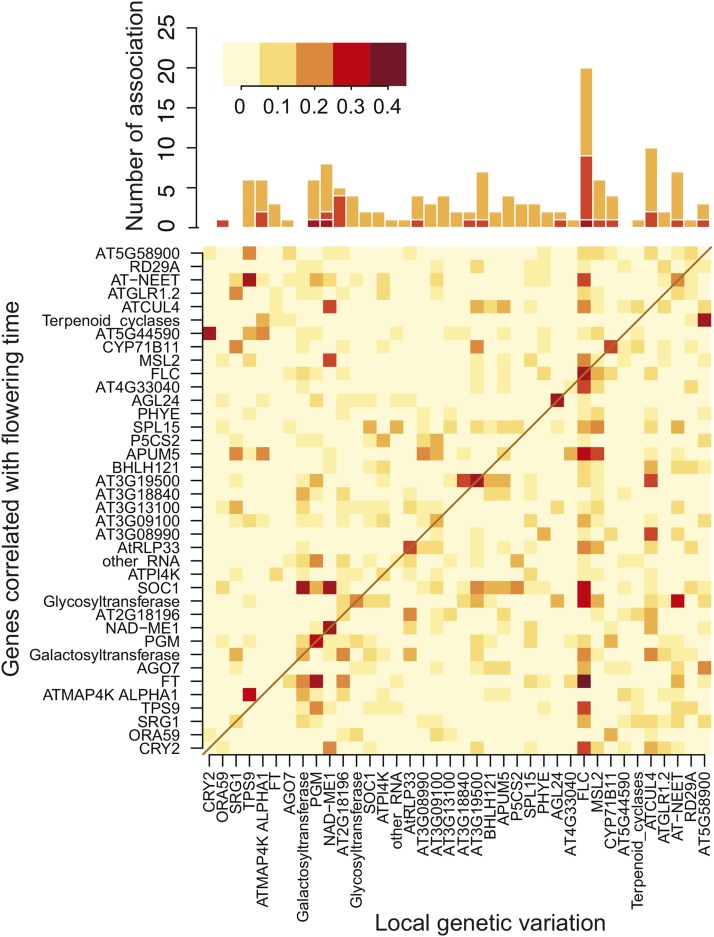
Genetic effects on gene expression levels. Effects of local genetic variation were estimated using a variance component analysis and 30-kb windows surrounding each gene in Table 1. The lower panel shows the fraction of expression variation explained by local genetic variation surrounding each gene (*cis*-effects are along the diagonal), and the top panel shows number of associations explaining more than 10% of the variation (*cf*. Table S2).

The correlation network between gene expression and flowering time (Figure 2C) inherently undirected and tells us little about causation, but insight can be gained by identifying the genetic causes of the expression variation (Schadt *et al.* 2005). We used variance component analysis (Lippert *et al.* 2014; Meng *et al.* 2016) to estimate the effect of local (*cis*-acting) genetic variation on gene expression, using a 30 kb window surrounding each gene. Based on permutation tests (p<0.05), roughly one third of the genes in Table 1 were significantly *cis*-regulated (Figure 3 and Table S2). *FLC* stood out in that not only it was strongly *cis*-regulated, but genetic variation at the gene was also significantly associated with expression of almost half of the other genes in Table 1. Thus genetic variation at *FLC* is affecting the expression of these loci in *trans*, almost certainly through its effect on *FLC* expression. In contrast, the expression level of several other genes highly correlated with flowering time, including *FT*, *SOC1*, and *CRY2* showed no evidence of *cis*-regulation, but strong evidence for being regulated by genetic variation at *FLC*. This suggests that *FLC* is the key determinant of flowering time under our conditions.

**Figure 4:**
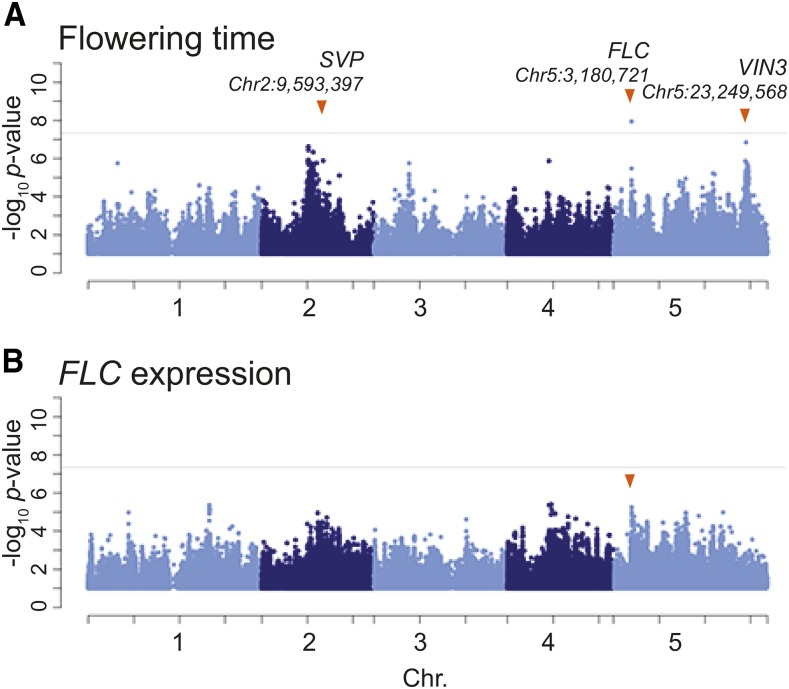
GWAS for flowering time (A) and *FLC* expression (B). Gray horizontal lines indicate Bonferroni-corrected 5% significance thresholds and orange arrows in panel A show *a priori* flowering time genes (from Sasaki *et al.* 2015); the arrow in B shows the SNP in the *FLC* region identified in A.

To further study the effect of *FLC*, we carried out genome-wide association studies (GWAS) for flowering time and *FLC* expression (Figures 4 and S1). In agreement with our previous results (Sasaki *et al.* 2015), GWAS for flowering time identified a genome-wide significant association with a single nucleotide polymorphism (SNP) in the promoter region of *FLC* (Chr5:3,180,721; *p*-value = 1.14E-08, MAF = 0.38) in addition to weaker associations in two other *a priori* candidates (Figure 4A). However, there was no significant association for *FLC* expression (Figure 4B), even at *FLC* itself — which is surprising given the strong correlation between *FLC* expression and flowering time (Table 1) and the evidence for *cis*-regulation obtained using variance-components analysis (Figure 3).

### A mediation model of flowering time variation

We are thus faced with a seemingly paradoxical result. How can a SNP at *FLC* (SNP*_FLC_*) predict flowering time but not *FLC* expression, when *FLC* expression strongly predicts flowering time (Figure 4)? We note that there is no non-synonymous variation in this gene (Li *et al.* 2014), so the effect of local genetic variation must be regulatory—and indeed the variance component analysis confirms the existence of massive *cis*-regulatory variation (Figure 3).

This suggests two things: first, SNP*_FLC_* must affect flowering through some aspect of *FLC* expression that is not captured by our expression data; second, the expression variation we measure must partly be caused by genetic variation not tagged by SNP*_FLC_* (in *cis* or in *trans*). As just noted, the variance-component analysis clearly supports *cis*-regulation of *FLC* (Figure 3).

**Figure 5:**
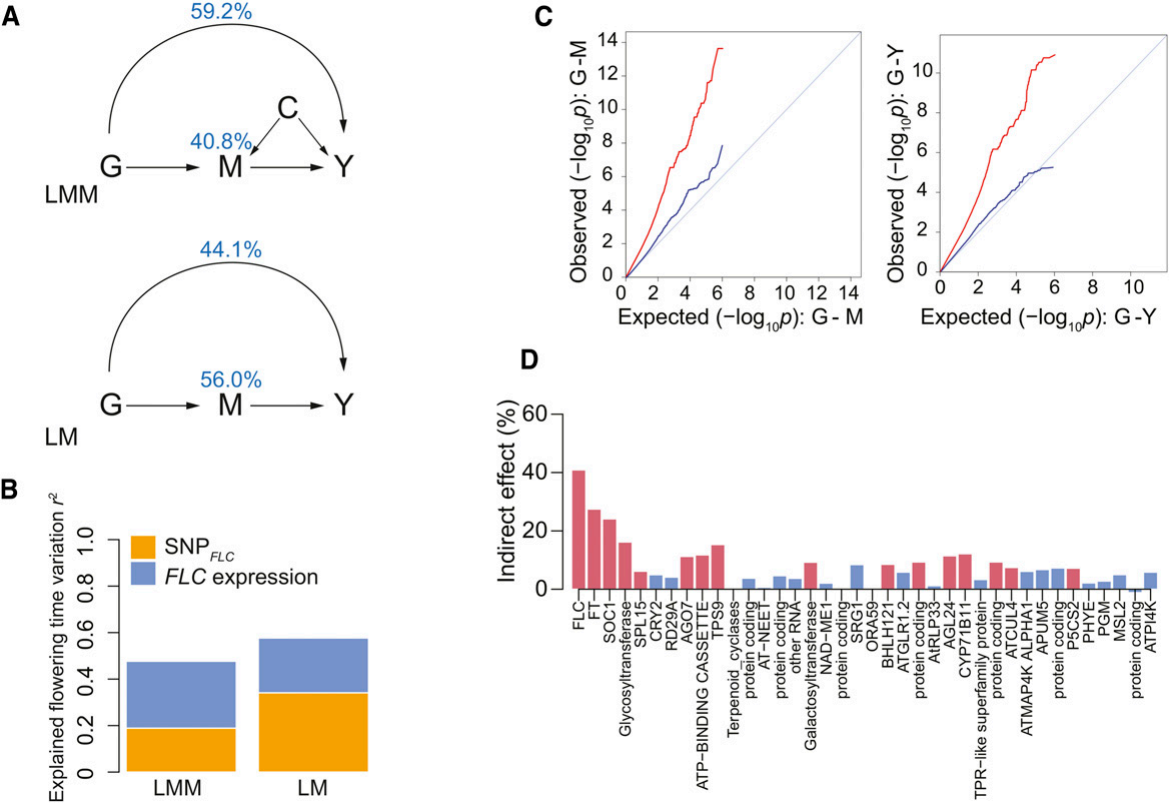
Mediation analysis of flowering time regulation by *FLC*. (A) Models used. The full model correcting for genetic background is shown on top (LMM, linear mixed-model), and the model without such a correction is shown below (LM, linear model). For details see text. Estimates are shown in blue. (B) Proportion of flowering time variation ($r^{2}$) explained by SNP*_FLC_* and *FLC* expression under the two models (see text). (C) QQ plots of genome-wide association for flowering time and *FLC* expression with (blue line) and without (red line) correcting for population structure. (D) The SNP*_FLC_* effect that is mediated by expression of each of the genes in Table 1. Red bars indicate that effect is significant (p<0.05).

To estimate the extent to which the effect of SNP*_FLC_* on flowering is captured by *FLC* expression, we performed a statistical mediation analysis (Baron 1986; Valeri and Vanderweele 2013; Palmer *et al.* 2017). Specifically, we modeled a trait Y under the regulation of a causal factor G that partly acts through an intermediate mediator M in the context of a confounding background factor C (Figures 1 and 5A). In the present context, we assumed that the SNP*_FLC_* (G) regulates flowering time (Y) and that this effect is partly captured by the measured *FLC* expression (M). Because *FLC* expression was measured at the vegetative stage, many weeks before flowering, we assume that M affects Y and not the other way around. We used a linear mixed-model approach (LMM; see Methods) to extend the mediation model to allow genetic background loci to affect both M and Y (Figure 5A).

Using this model, we estimate that 40.8% of the total effect of SNP*_FLC_* is mediated by (measured) *FLC* expression. As argued above, the remaining 59.2% must thus be due to unmeasured effects on *FLC* regulation, as it is hard to see how SNP*_FLC_* could affect flowering any other way.

Furthermore, the model explained nearly half of the phenotypic variation ($r^{2}=0.48$), and both SNP*_FLC_* and *FLC* expression contributed significantly (p<0.01). Interestingly, the latter explained more of the variation ($r^{2}=0.29$) than the former ($r^{2}=0.19$), presumably reflecting *cis*-genetic variation at *FLC* not tagged by SNP*_FLC_* as well as the effect of *trans*-acting background genetic loci (Figure 5B).

The importance of the genetic background can readily be seen by comparing the result above to those obtained using a model that does not control for confounding genetic background (Figure 5A). Under this model, SNP*_FLC_* explained a much higher proportion of the phenotypic variance ($r^{2}=0.34$ *vs.* $r^{2}=0.19$ above), as observed in the presence of confounding (Figure 5B). The effect of confounding can also be seen in a genome-wide inflation of *p*-values (Figure 5C). Finally, we investigated the extent to which the effect of SNP*_FLC_* might be mediated by the expression levels of other genes by simply replacing *FLC* expression with that of another gene in the model (Figure 5A). Of the 38 genes in Table 1, 16 showed significant mediation of SNP*_FLC_* at p≤0.05 (Figure 5D). Among those were most of the genes having flowering-related mutant phenotypes. Correlation with *FLC* expression was not a strong predictor for mediating the SNP*_FLC_* effect. For example, genes related to the integrator pathway, including *FT*, *SOC1*, *AGL24*, and *SPL15* (Figure 2), all mediated SNP*_FLC_* regardless of the correlation with *FLC* expression. On the other hand, *CRY2* in the photoperiod pathway did not mediate SNP*_FLC_* although its expression is significantly correlated with that of *FLC*. In contradiction to this result, the variance component analysis shows *trans* regulation of *FLC* on *CRY2* expression (Figure 3). These suggest that *CRY2* might be regulated by *FLC* polymorphisms not tagged by SNP*_FLC_* (or epistasis).

### Prediction of flowering time using the *FLC* model

**Figure 6:**
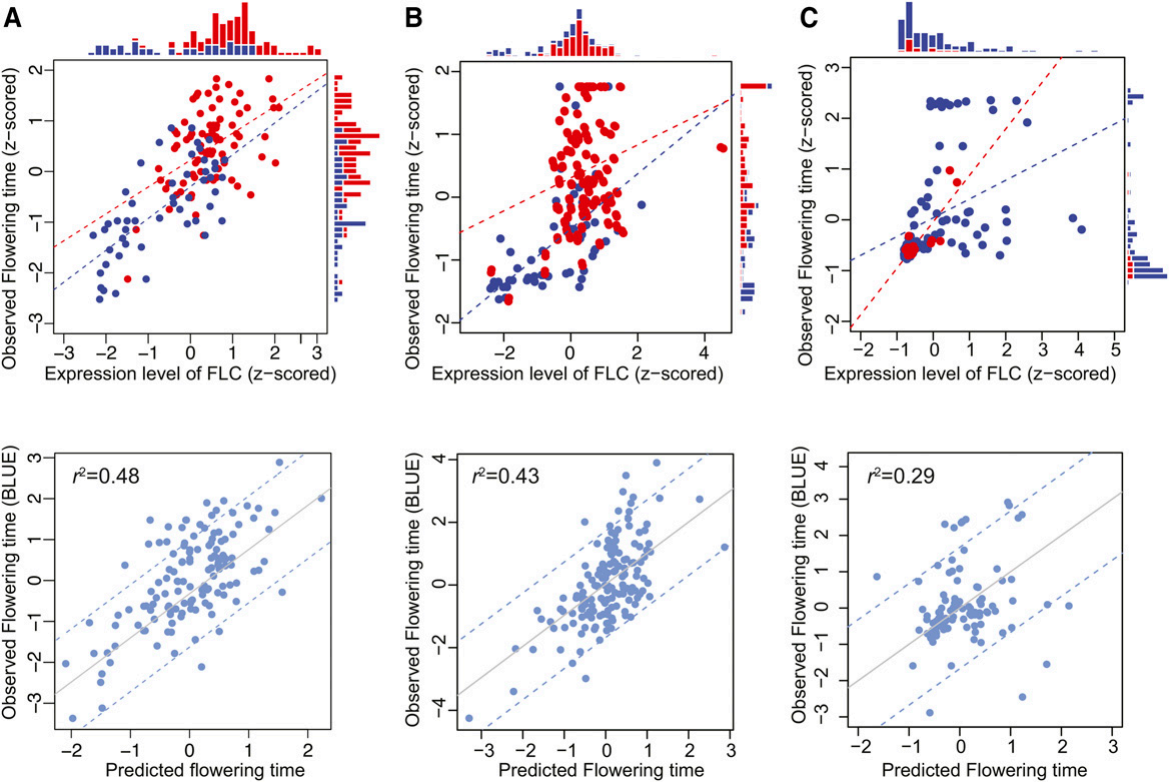
Prediction of flowering time. (A) Top: A scatter plot between flowering time and the expression level of *FLC*, both at 10°C, with histograms for each phenotype illustrating the effect of SNP*_FLC_*. Reference and non-reference alleles are shown in blue and red, respectively. The dashed lines are regression lines for each allele. Bottom: predicted *vs.* observed flowering time. (B) The 10°C model applied to the same population grown at 16°C. (C) The 10°C model applied to a different population grown in the greenhouse. Dashed lines in model fits show 95% confidence intervals.

As described in the previous section, we explain almost half of flowering time variation ($r^{2}=0.48$) using SNP*_FLC_* ($r^{2}=0.19$) and SNP*_FLC_*-independent *FLC* expression ($r^{2}=0.29$). Thus a single SNP and a single expression measurement allows us to predict flowering time rather well (Figure 6A).

To investigate the limits of this “single gene” model we tried to predict flowering using flowering time and expression data generated for the same population, but at a higher growth temperature, namely 16°C (Dubin *et al.* 2015). Higher temperature generally accelerates flowering, but also prevents vernalization (Duncan *et al.* 2015), thus significantly delaying flowering for some genotypes (Sasaki *et al.* 2015).

We predicted flowering time at 16°C using SNP*_FLC_* and *FLC* expression at 16°C with effects of genetic background. We applied parameters estimated using the 10°C data to the model (see Methods for details). SNP*_FLC_* was significantly associated with flowering time in the 16°C data as well (*p*-value = 3.31E-07; Figure S1A-B), but a correlation between *FLC* expression and flowering time was only seen for early-flowering lines that have no requirement of vernalization (*cf*. Figures 6A and B). Nonetheless the performance of the model changed surprisingly little (the explained variation of flowering time decreased from 48 to 43% (Figure 6B).

We also tested the model on a different population for which greenhouse (approximately 23°C) and *FLC* expression data were available (Shindo *et al.* 2005). In these data, SNP*_FLC_* was not significantly associated with flowering time, but *FLC* expression still showed a weak correlation with flowering, and the model predicted 29% of flowering time variation (Figure 6C). This decreased prediction accuracy might be due to unknown genes that affect *FLC* action as suggested by Shindo *et al.* (2005).

## Discussion

Our primary goal in this study was to use flowering time in *A. thaliana* as a test case for understanding the connection between genotype and phenotype. Specifically, we built a statistical model to understand how genetic variation and gene expression variation at the central flowering regulator *FLC* combine to cause phenotypic variation. Both variables are significantly correlated with flowering time, but not with each other. We resolve this apparent paradox by demonstrating that genetic variation at *FLC* is only partly captured by measuring *FLC* expression, and that *FLC* expression also captures the effect of genetic background loci. The complexity apparent in even such a simple network has broader implications for our ability to understanding the genotype-phenotype map. We also demonstrate that it is essential to control for genetic background in these kinds of studies. Using a classical linear mixed model (LMM) approach commonly used in GWAS studies, we developed a simple mediation model that takes genetic background into account, and showed that it dramatically reduced overestimation of the effect of *FLC*. Although Principal Component Analysis (PCA) can, in principle, also handle complex confounding (Yang *et al.* 2017), the LMM-based approach is simple, and has a clear theoretical justification and interpretation (Vilhjálmsson and Nordborg 2013).

According to our estimates, less than half of the effect of the main SNP at *FLC* is captured by *FLC* expression (Figure 5). Given that there is no non-synonymous variation at *FLC*, the missing variation must reflect aspects of *FLC* expression we did not measure (*e.g.*, tissue- or time-specific expression). Conversely, the fact that *FLC* expression only partly reflects the main SNP almost certainly reflects both allelic heterogeneity at *FLC* (Hagenblad *et al.* 2004; Shindo *et al.* 2005; Li *et al.* 2014) and background genetic loci. Integration analyses have reported weak connection in genetic regulation between intermediate and final phenotypes in both *A. thaliana* and humans (Zhang *et al.* 2011; GTExConsortium 2017). Although the observation has been attributed to noise and other confounding effects (Leek and Storey 2007; Fusi *et al.* 2012), genetic complexity likely also contributes. Mediation analyses like those carried out here should help resolve this.

Our results also shed some light on the network regulating flowering time. Our correlation and variance component analyses (Figures 2–3), support the considerable experimental evidence that *FLC* works upstream of the integration and photoperiod pathways, controlling the expression of key flowering time genes like *FT* and *SOC1* in the integration pathway and *CRY2* in the photoperiod pathway (Hepworth *et al.* 2002; El-Assal *et al.* 2003; Michaels *et al.* 2005). However, it is interesting to note that the effect of SNP*_FLC_* was mediated by *FT* and *SOC1* but not *CRY2* (Figure 5D). This suggests that *FLC* may regulate these pathways differently. In general, however, the central role played by *FLC* is illustrated by how well our simple model predicts flowering time across populations and environments (Figure 6).

In conclusion, our results illustrate how genetic variation and intermediate phenotypes such as gene expression may be combined to understand the genotype-phenotype map, while at the same time illustrating the complexity of even an extremely simple network dominated by a single locus.
